# Association between Changes in Nutrient Intake and Changes in Muscle Strength and Physical Performance in the SarcoPhAge Cohort

**DOI:** 10.3390/nu12113485

**Published:** 2020-11-13

**Authors:** Laetitia Lengelé, Pauline Moehlinger, Olivier Bruyère, Médéa Locquet, Jean-Yves Reginster, Charlotte Beaudart

**Affiliations:** 1 WHO Collaborating Centre for Public Health Aspects of Musculoskeletal Health and Aging, Division of Public Health, Epidemiology and Health Economics, University of Liège, CHU—Sart Tilman, Quartier Hôpital, Avenue Hippocrate 13 (Bât. B23), 4000 Liège, Belgium; llengele@uliege.be (L.L.); medea.locquet@uliege.be (M.L.); jyr.ch@bluewin.ch (J.-Y.R.); c.beaudart@uliege.be (C.B.); 2 Engineering and Health Field: People, Bioproducts, Environment, Paris Institute of Technology for Life, Food and Environmental Sciences, 16 Claude Bernard Street, CEDEX 05, F-75231 Paris, France; pauline.moehlinger@agroparistech.fr; 3 Chair for Biomarkers of Chronic Diseases, Biochemistry Department, College of Science, King Saud. University, Riyadh 11451, Saudi Arabia

**Keywords:** malnutrition, SarcoPhAge, macronutrients, micronutrients, muscle strength, physical performance, gait speed

## Abstract

Muscle weakness and physical performance impairment are common geriatric conditions that raise morbidity and mortality. They are known to be affected by nutrition, but only a few longitudinal studies exist. This study aims to fill this gap by exploring the association, over 3 years, between variations of nutrient intakes, as well as, on one side, the variations of handgrip strength, as a surrogate of muscle strength, and on the other side, the physical performance, assessed by gait speed. Participants from the SarcoPhAge study, a Belgian cohort of people aged 65 years and older, were asked to complete a self-administered food frequency questionnaire (FFQ) at the second (T2) and the fifth (T5) year of follow-up. Daily macro- and micronutrient intakes were measured and their changes in consumption over the three years of follow-up were then calculated. The association between changes in nutrients consumption and the variations in muscle parameters were investigated through multiple linear regressions. Out of the 534 participants included in the cohort, 238 had complete data at T2 and T5 (median age of 72.0 years (70.0–78.0 years), 60.9% women). In the cross-sectional analysis, calories, omega-3 fatty acids, potassium, and vitamins D, A, and K intakes were positively correlated with muscle strength. In the longitudinal analysis, neither the gait speed nor the muscle strength changes were significantly impacted by the variations. Other longitudinal investigations with longer follow-up are required to improve knowledge about these interrelations.

## 1. Introduction

Muscle function decline and impaired physical performance characterize, among others factors, the aging process [1,2]. These two parameters are significant indicators of muscle health as they are part of the definition of sarcopenia, a common muscle disease in older adults defined by a low muscle mass, a reduction in muscle strength, and/or a decrease in physical performance [3]. The age-associated decline in muscle strength is more rapid than muscle mass loss, with an annual rate decline varying from 1.9% to 5.0% [4,5], compared to a maximum of 1% for muscle mass in older adults [5]. Although the decline in muscle strength is associated with the decline in muscle mass in older adults, maintaining or increasing muscle mass does not prevent the loss of muscle strength [5]. Regarding the measure of the gait speed, it is a major indicator of the independence level of older adults in daily activities [6] and, therefore, provides a good predictive value for the onset of disability. 

These two muscle health components are associated with real public health challenges. Indeed, their deterioration increases the risk of health-related adverse consequences such as longer hospital stays, higher risk of institutionalizations and falls, lower quality of life, and increased mortality [7,8,9,10,11,12,13,14]. In terms of costs, it is recognized that all these consequences significantly increase health care costs both for society and the patient [15,16]. The age-associated muscle wasting disorders are impacted by multiple factors, including biological (i.e., hormones, inflammation, insulin resistance), psychosocial (i.e., self-efficacy, fear of falling), and lifestyle factors like nutrition and exercise [17]. Since the lifestyle factors are modifiable, research focusing on these is essential to help to improve strategies in the prevention and treatment of impaired function and disabilities.

Malnutrition accentuates age-related physical function loss [18,19], furthermore raising disability, morbidity, and mortality [20,21,22]. It is frequent for older individuals to experience a loss of appetite and therefore decrease their food consumption [23,24,25]. This condition, called anorexia of aging, has multiple determinants including medical, social, environmental, and psychological factors [24,25,26,27]. The altered eating habits affect the amount of food intake, as older adults consume from 16% to 20% lower calories than younger adults [26,28], and worsen the risk of nutrient inadequacy in older adults [29]. There is now evidence that links nutrition to muscle health parameters [18,30,31,32,33], highlighting the relevance of appropriate nutritional strategies to limit the decline of muscle strength and physical performance. However, the literature mostly includes cross-sectional studies with inconsistent results. Indeed, some studies provide evidence that higher nutrient intake benefits muscle health, while others provide no findings [33]. A recent study, performed in the sarcopenia and physical impairment with advancing age (SarcoPhAge) cohort, has indicated a cross-sectional association between low nutrient consumption and sarcopenia [34].

Longitudinal analyses would be required to investigate the impact of dietary changes on muscle parameter changes. Longitudinal studies exist, such as those on protein [35,36], vitamin D [37,38], C, and E intakes [39], but they only measure the longitudinal change in muscle parameters according to baseline dietary intakes, and do not measure the change in dietary intakes during follow-up. This is important because there is a decline in energy consumption with advancing age that can compromise nutrient intake in older adults [40], due to several reasons mentioned above, especially regarding anorexia of aging. Additionally, the absorption of nutrients decreases with age [41]. Given the fact that dietary intake varies broadly with increasing age, measuring the change in dietary intake during the follow-up of the study therefore appears essential to establish a causal relationship with the decline in muscle health. This is exactly the aim of the present longitudinal study: To explore the effect of variations of macro- and micronutrient intakes on muscle parameters changes, specifically muscle strength and physical performance, in the SarcoPhAge cohort. 

## 2. Materials and Methods

### 2.1. Participants’ Characteristics

Participants from the SarcoPhAge study were included in the present analysis. The full methodology and protocol of the SarcoPhAge study have already been described in detail previously [34]. Briefly, the SarcoPhAge study is an observational study, which included 534 older adults in Liège, Belgium, followed up from June 2013 to September 2019. The cohort includes community-dwelling adults aged 65 years or older with an annual follow-up. No specific exclusion criteria related to health or demographic characteristics were applied, except for the exclusion criteria established for the Dual-energy X-ray Absorptiometry scan for individuals with an amputated limb or with a body mass index (BMI) above 50 kg/m^2^. Written informed consent was provided by participants, and the study was approved by the ethics committee of our institution (reference 2012/277), with two amendments in 2015 and 2018. 

During the second year of follow-up of the participants (T2), a food frequency questionnaire (FFQ) was self-administered to assess the macro- and micronutrient intakes of the participants. Although the questionnaire has not been validated in an older population, it has been developed by a group of experts in the field. The FFQ was self-administered for a second time during the last year of follow-up (T5). Thus, we have two full sets of data, separated by three years, which offer the possibility of prospective analyses. The present longitudinal study is based on the population still participating in the SarcoPhAge study at T5 and who completed the FFQs at both T2 and T5. Among the 534 participants included in the SarcoPhAge study, 238 met this condition. 

### 2.2. Data Collection

Participants were seen in the Polyclinique Lucien Brull in Liège, Belgium, by one research assistant for a mean time of 1 h. During their follow-up visits, among a series of tests and evaluations, their muscle health was evaluated and they were asked to complete an FFQ. 

### 2.3. Assessment of Physical and Muscle Parameters

Physical performance was assessed by the usual gait speed on a 4-m distance [42]. Muscle function was assessed by muscle strength using the hand-held hydraulic dynamometer (Saehan Corporation—MSD Europe BVBA, Belgium), calibrated each year for 10, 40, and 90 kg. For this test, participants had to squeeze the device as hard as possible, three times with each hand. The highest measure was recorded, as advised by the Southampton protocol [43].

### 2.4. Energy and Nutrient Intakes

The complete methodology and protocol for the treatment of FFQs and their analyses have already been described in detail elsewhere [34]. The same methodology of processing FFQs was applied for T2 and for T5, which is briefly recalled here. 

Each participant was asked to complete a self-administered FFQ to assess their usual food intake over one month and to bring this completed questionnaire at the T2 and T5 follow-up visits. For each food in the FFQ, the daily amount consumed was calculated according to the following formula:

Quantity consumed = Frequency × portion size

The quantity consumed, calculated from the FFQ data, was expressed in g/day for solid food and in mL/day for drinks. For non-standardized portions, a list of images representing seven different sizes of portions of various food was used. Participants had to choose between them the image that best represented, in their opinion, the size of their portions. Then, for each of the food items of the FFQ, a detailed nutrient composition was calculated using the NutriNet-Santé table [44]. This food composition table is used in the NutriNet-Santé study, a large prospective cohort followed across 10 years to collect information on nutrition and health. When items grouped several foods, such as the low-fat cheese item which includes several kinds of cheese, the nutritional composition was calculated as the mean composition of all the corresponding foods from the NutriNet-Santé table. The mean composition was then weighted by the frequency of sex-specific consumption of each food among the older participants of the NutriNet-Santé study. We then measured the total energy intake, and the consumption of micro- and macronutrients per day and per participant. The following macronutrients were selected for the study: Proteins, lipids, saturated fatty acids (AGS), polyunsaturated fatty acids (AGP), omega 3 and 6 fatty acids, monounsaturated fatty acids (AGM), and carbohydrates. For micronutrients, sodium, potassium, magnesium, phosphorus, iron, calcium, zinc, and vitamins D, A, E, C, and K were studied. 

### 2.5. Covariate Data Collection

In this study, we also used the following information that had been collected for all participants during the annual follow-ups: Age, sex, body mass index (BMI), smoking status (yes/no), number of comorbidities, number of drugs consumed, and the level of physical activity based on the Minnesota leisure time activity questionnaire [45]. These covariates are known to significantly affect muscle health and dietary intake [46,47,48,49,50]. Furthermore, they have been identified as such in previous studies of the SarcoPhAge cohort [51].

### 2.6. Statistical Analyses

The data were processed using the SPSS Statistics 24 (IBM Corporation, Armonk, NY, USA) software package. The normality of the variables was checked by examining the histogram, the quantile–quantile plot, the Shapiro–Wilk test, and the difference between the mean and the median values. The characteristics of the population and the consumption of micronutrients and macronutrients were expressed as median (twenty-fifth to seventy-fifth percentile) because they did not follow a Gaussian distribution. Binary and categorical variables were described by absolute (*n*) and relative (%) frequencies. A global evaluation of all participants’ baseline characteristics was performed. 

Differences in socio-demographics and clinical characteristics, and in the consumption of micronutrients and macronutrients between T2 and T5 were investigated through Wilcoxon paired tests for skewed variables. Both the associations between nutrient consumption and either muscle strength or gait speed at T2 were studied by multiple linear regressions with one of the muscle parameters as a dependent variable and each nutrient as independent variable. The multiple covariates that were incorporated into the model are shown here at the bottom of each of the corresponding tables. In order to study the association between the evolution of nutrient consumption and the evolution of muscle parameters between T2 and T5, multiple linear regressions were performed including the confounding variables presented above. The evolution of muscle parameters was calculated by the absolute difference between the variables at T2 and T5. To calculate the evolution of nutritional parameters, macronutrient intakes were calculated as a percentage relative to the total caloric intake of the participant and for micronutrients, the amount was expressed per 1000 kcal. Both macro- and micronutrient changes correspond to the difference between T2 and T5. This method corresponds to an adjustment method called the density method. It uses the quotient of the nutrient intake over the total energy intake [52]. Indeed, because of the strong correlation between nutritional intake and caloric intake, it was essential to adjust the nutrient intake on the energy intake if we wanted to know the isolated effect of each nutrient. Other adjustment methods could have been applied, but the density method appeared to be the most easily interpretable, and nutrient densities are used in the Belgian national nutritional recommendations [53]. 

Sensibility analyses were performed to assess the robustness of our results, with the relative difference of muscle parameters between T2 and T5 instead of the absolute difference. The relative difference was obtained by dividing the absolute difference by the initial value (at T2) of the parameter. 

The results were considered statistically significant at the 0.05 critical threshold.

## 3. Results

### 3.1. Characteristics of Participants

**Figure 1:**
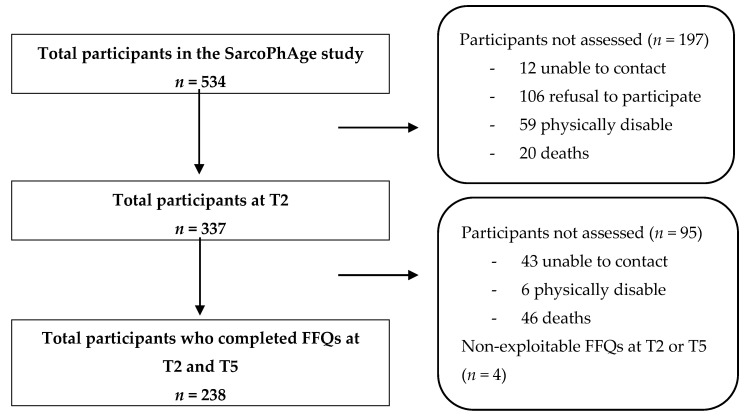
Flow chart.

Out of the 534 older adults initially included in the SarcoPhAge study, a total of 238 individuals completed both FFQs at T2 and T5 and were included in the present study (median age of 72 years (70.0–78.0), 60.9% of women) (Figure 1). Sociodemographic and clinical characteristics of the whole population are presented in Table 1. As a summary, participants had good cognitive status (mini-mental state examination (MMSE) >24 points), a median of 4 concomitant diseases at T2 and T5, consumed daily 6 drugs at T2 and 7 drugs at T5, and 7.6% (*n* = 18) were smokers. The level of physical activity increased between the two time points for both men and women (*p* < 0.001), and the body mass index significantly decreased (*p* = 0.01). Included participants were compared to the patients who did not complete the FFQ to investigate the potential differences. Lost-to-follow-up patients were older (median age of 74.6 (69.6–79.7) vs. 70.4 (67.5–75.3), *p* ≤0.001), had lower muscle strength (median of 25.0 (18.0–35.0) vs. 28.0 kg (22.0–39.0) *p* ≤0.001) and were composed of more malnourished patients according to the mini-nutritional assessment questionnaire (18.5% vs. 9.3%, *p* = 0.003) than patients included in this study.

### 3.2. Dietary Nutrient Consumption

The nutritional intakes of the population at T2 and T5 are displayed in Table 2. Overall, FFQ analyses revealed that participants had a lower total energy intake at T5 than at T2 (1615.4 versus 1767.9 kcal/day, *p*-value = 0.002). Regarding macronutrients, the volunteers at T5 consumed less carbohydrates than at T2 (32.68% at T5 and 34.76% at T2, difference of −2%, *p*-value < 0.001). The participants at T5 also consumed more saturated fatty acids than at T2 (+0.53%, *p*-value = 0.038). The following micronutrients were significantly less consumed by participants at T5 than at T2: Sodium, magnesium, iron, calcium, and zinc (all *p*-values < 0.05). No other significant difference between the consumption of micro- and macronutrients between T2 and T5 were observed. Absolute consumption of these macro- and micronutrients (in terms of quantity/day) is presented in the Appendix A Table A1).

Moreover, less than half of the participants met the Belgian national recommendations for the carbohydrates, saturated fatty acids, omega-3 and omega-6 fatty acids, vitamins D, C, and K, sodium, and potassium (Table A3) [53].

### 3.3. Association between Macro- and Micronutrients and Muscle Health Components

From the cross-sectional analyses on our baseline population (Table 3) at T2, it emerged that muscle strength seemed to be positively associated, after adjustment for potential confounding variables, with calorie intake (*p* = 0.003) and the consumption of omega-3 fatty acids (*p* = 0.03), potassium (*p* = 0.04), and vitamins D (*p* = 0.03), A (*p* = 0.045), and K (*p* = 0.01). 

When we carried out further longitudinal analyses to assess the changes of nutritional consumption between T2 and T5 and their effect on changes of muscle parameters during the same period (Table 4), no association was found to be statistically significant between either changes in gait speed or muscle strength and changes in dietary intakes.

In the sensitivity analysis, when we performed similar longitudinal analyses but with the relative difference of muscle parameters between T2 and T5 instead of the absolute difference, we found similar conclusions except for one nutrient: An increase in saturated fatty acids seemed to have a positive impact on the muscle strength evolution (*p* = 0.039) (Table A2 in the Appendix A).

## 4. Discussion

At the baseline, a higher consumption of calories, omega-3 fatty acids, potassium, and vitamins D, A, and K seemed to be positively associated with better muscle strength. However, when we analyzed the impact of the changes in nutrient intake across years on the muscle parameters changes, no nutrient was correlated with changes of gait speed or muscle strength between the baseline and the three-year follow-up. In our older population from the SarcoPhAge cohort, the absolute dietary intake had significantly decreased for almost every macro- and micronutrient over a period of three years (i.e., between T2 and T5). This was in line with the fact that the older adults from our cohort experienced a deficit in food consumption also called “anorexia of aging” [26]. People suffering from this condition are at high risk of protein-energy malnutrition, sarcopenia, and frailty, leading to higher morbidity and mortality [26,54]. Furthermore, adjusted on the calorie intake, the amount of carbohydrates, sodium, magnesium, iron, calcium, and zinc taken by the population of our sample has significantly declined. When we compared dietary intakes to Belgian national recommendations (Table A3) [53], less than half of the participants met these recommendations for the carbohydrates, saturated fatty acids, omega-3 and omega-6 fatty acids, vitamins D, C, and K, sodium, and potassium. Regarding the muscle parameters, both muscle strength and gait speed significantly decreased at the end of the three-year follow-up. The median decline in muscle strength after three years of follow-up reached the minimal clinical important difference (MCID) value ranged between 5.0 and 6.5 kg [55], but the median difference in gait speed between the two time points did not reach the MCID value of 1.0 m/s [56]. This could have impacted our analyses because this change in gait speed would have been too small to detect an influence of dietary intake on it.

Several studies, mentioned below, have already investigated the cross-sectional impact of macro- and micronutrients on muscle parameters. Some of them, like our study, investigated the dietary intake alone, while others studied the biochemical status with or without the nutrient consumption. Two reviews focusing on the relationship between muscle strength and, either or both, the biochemical status of nutrients and the dietary intake, corroborate our results regarding omega-3 fatty acids. Indeed, they concluded that omega-3 fatty acids were positively correlated to muscle strength among older adults in cross-sectional studies [57,58]. Regarding vitamin A, one of these reviews discussed the carotenoid status, where a lower blood concentration of carotenoids was associated with lower muscle strength in cross-sectional analyses including older adults [57]. Moreover, the carotenoid status was found to have a long-term impact on muscle strength in the InCHIANTI study, where older community-dwelling adults with lower plasma carotenoids levels were at higher risk of low grip strength (OR = 1.88, 95% CI, 0.93–3.56, *p* = 0.07) [59]. The anti-inflammatory and antioxidant potentials of these two nutrients, vitamin A and omega-3 fatty acids, could explain the effects observed [60,61]. Regarding vitamin D, several studies have investigated the biochemical status, the 25-hydroxyvitamin D blood concentration, and its association with muscle strength and physical performance in older adults. A first study of Houston et al. found a positive association between a low serum concentration of vitamin D and low handgrip strength (*p* < 0.05) and with poor physical performance too, measured by the short physical performance battery test (*p* < 0.05) [62]. While these results are in line with ours concerning the association with muscle strength, it is contrasting with our results concerning physical performance. These cross-sectional results were confirmed in a second study of Houston et al., and longitudinal associations were also explored in this study, where patients with a low blood serum concentration of 25-hydroxyvitamin D at the baseline had poorer physical performance at 2 and 4 years of follow-up (*p* < 0.01) but not lower grip strength (*p* > 0.05) [38]. Inconsistent longitudinal results were found in the study by Visser et al. regarding muscle strength, where participants with a low blood concentration of vitamin D at the baseline had 2.57 (95% CI 1.40–4.70) more risk of experiencing low muscle strength [37]. Evidence suggests that this vitamin can stimulate the proliferation and the differentiation of the skeletal muscle fibers, thus enhancing muscle strength [63]. Concerning vitamin K, one study, including 1089 community-dwelling older adults and investigating the biochemical status of this vitamin, found a statistically significant positive association both with muscle strength (*p* < 0.04) and gait speed cross-sectionally [64]. While these results are in line with ours concerning the association with muscle strength, it is contrasting with our results concerning gait speed. Nowadays, the role of vitamin K on muscle health is not yet fully understood, and more studies on its biological mechanisms and its impact on muscle function are needed [65]. To our knowledge, no research investigating the link between potassium and muscle strength has been performed, except for the study of Beaudart et al. in the same cohort as the one of the present study, where potassium intake was associated with lower risk of sarcopenia (*p* = 0.04) [34]. Potassium is necessary for nerve activity and therefore contributes to the contractibility of the muscle [66].

From a longitudinal perspective, we cannot confirm the relationships observed in the cross-sectional analyses. In fact, the muscle parameters changes did not seem to be impacted by the nutrient intake changes during the three years of follow-up. Several possible hypotheses could explain the different results between the cross-sectional and longitudinal analyses. Firstly, the length of follow-up, potentially too short, could have impacted the statistical power of our study and secondly, we can mention the biases inherent to the dietary assessment method like the recall bias. Moreover, these are two different investigations. Indeed, the cross-sectional analyses evaluated a precise value at a given time, while the longitudinal analyses measured a difference between T2 and T5. Therefore, the results and the conclusions are difficult to compare adequately. To our knowledge, no study exists on the longitudinal association between changes in dietary intake and changes in muscle parameters, in older adults aged over 65 years. Longitudinal research on this topic only studied food consumption at baseline and its impact on the muscle strength and physical performance changes over time. Therefore, we cannot compare our results regarding the longitudinal analysis since the nutritional data evaluated were not similar. Moreover, our conclusions were confirmed by the assessment of the robustness of our results, which were identical to the analyses performed on the relative variations instead of the absolute variations of muscle parameters. Only the saturated fatty acids became significantly positively associated with muscle strength. Yet, this relationship has not been investigated elsewhere. There is a real lack of research on the longitudinal effects of nutrient intake changes on muscle parameter changes. Nonetheless, food consumption of older adults can vary broadly over a period of only three years as described in this present study. This type of longitudinal research is therefore essential.

### Strengths and Limitations

This study has an original design. It is one of the first studies to consider longitudinal changes in the intake of a large number of nutrients and to evaluate how those changes impact individual muscle parameter changes. Another strength of this study is the adjustment of the macro- and micronutrient intakes on the total energy intake according to the density method. This allowed us to avoid the impact of any existing correlation between the consumption of calories and nutrients on our results. 

Several aspects must be taken into consideration when interpreting our results. Firstly, we measured the dietary intake but not the biochemical status of macro- and micronutrients. Dynamic factors could alter single nutrient absorption, when consumed with other nutrients [29], such as the known synergy between vitamin D and calcium. Moreover, the biochemical status of some micronutrients, such as calcium and magnesium, is complex to evaluate because they have no specific markers [29]. Secondly, we did not take into account the potential impact of a more global diet. In fact, we studied the impact of each specific nutrient, but it is a necessary first step before considering the overall nutritional quality. In addition, we adjusted our analyses on a large number of covariates, known to affect dietary intake and the muscle parameters, but other confounding factors could have been considered. Indeed, we took into account the level of physical activity of the participants but not the type of physical activity. It is now well established that resistance exercise training and aerobic exercise training are two types of exercise that have a positive effect on muscle and even help prevent a decline in mobility [67]. These exercises may enhance the myofibrillar protein synthesis, and it has been suggested that it was due to nutrient-stimulated vasodilatation and improvement in nutrient delivery to muscle [68]. Additionally, the results indicated that the effects of these types of physical activity, particularly resistance training, may be impacted by nutritional status [69] and nutrient supplementation [70]. Ethnicity is also known to influence muscle parameters and nutritional status [71,72], but we did not adjust our analyses on this variable since the included participants in our cohort were homogeneous in terms of ethnicity. The probability that this could predominantly have impacted our results remains low.

The FFQ entailed an inevitable reporting bias in the data reporting since it is based on participants’ memory. This dietary assessment method was chosen for the following reasons: It does not require trained interviewers, it can be self-administrated, and it can be used for large scale studies. Nevertheless, other methods to record daily dietary intake are available and could have been used. Moreover, a selection bias was brought by the constitution of our population that was composed of volunteers. Indeed, they were presumably in better health than the general population, and the evolution of their muscle parameters was therefore potentially better. Additionally, the decline observed for gait speed did not even reach the small detectable change threshold of 0.05 m/s [56], which could eventually partially explain why we did not find any association between nutrient consumption and gait speed. There was also a potential attrition bias because of the patients lost to follow-up. They possibly underwent a more important health decline than the participants interviewed throughout the entire follow-up. In fact, as presented in the results section, the volunteers included in the study had a better muscle strength and physical performance, were younger, and were composed of less malnourished patients than those lost to follow-up. Consequently, our results are probably not truly representative of the target population and they cannot be generalized to other populations or geriatric settings. Finally, we did not estimate one potential confounding factor, which is the consumption of nutritional supplements among participants. The intake of any supplemented nutrients could have been increased without being assessed in our analyses, and thus have an indirect positive impact on the muscle components, affecting the potential significant observations in our models. However, these data were not available. 

## 5. Conclusions

Based on an FFQ dietary assessment method, muscle strength seems positively associated with caloric intake, omega-3 fatty acids, potassium, and vitamin D, A, and K consumption at a given time. When studying variations over a period of three years, no association was found between the evolution of nutrient intake and either gait speed or muscle strength. The longitudinal impact of dietary intake on muscle parameters needs further investigation to fill the gap in the current knowledge on this subject. Cohort studies with a longer follow-up and longitudinal investigations on dietary patterns and their impact on muscle health are needed to elaborate on our findings. It is important to better understand these interrelations to enable the implementation of optimal nutritional strategies for the prevention of age-related muscle disabilities. 

## Figures and Tables

**Table 1:** Socio-demographic and clinical characteristics of participants at inclusion (T2) and after three years of follow-up (T5).

	T2 (*n* = 238)	T5 (*n* = 238)	*p*-Value
**Age (years)**	72.0 (70.0–78.0)	76 (73.0–81.0)	<0.001
**Sex**			
Women	145 (60.9)	145 (60.9)	---
**Number of drugs**	6.0 (4.0–8.0)	7.0 (5.0–10.0)	<0.001
**Number of concomitant diseases**	4.0 (2.7–5.0)	4.0 (3.0–5.0)	0.001
**MMSE (/30 points)**			
25–30 points	231 (97.1)	222 (93.3)	0.04
21–24 points	2 (0.8)	13 (5.5)	
≤20 points	3 (1.2)	2 (0.8)	
**Level of physical activity (kcal/day)**			
Women	1323.0 (677.2–2527.2)	1484.5 (847.0–2697.0)	<0.001
Men	1687.0 (1011.5–2761.7)	1837.0 (847.0–3265.9)	<0.001
**Smoking**			
Yes	18 (7.6) *	18 (7.6) *	---
**Body mass index (kg/m^2^)**	26.9 (23.9–29.4)	26.4 (23.6–29.5)	0.01
**Gait speed (m/s)**	1.2 (1.0–1.3)	1.2 (1.0–1.4)	0.01
**Grip strength (kg)**			
Women	21.0 (18.0–24.0)	16.0 (12.0–18.5)	<0.001
Men	39.5 (34.5–44.0)	32.0 (26.5–39.0)	<0.001

Quantitative variables were expressed as median (twenty-fifth to seventy-fifth percentile), and binary or categorical variables were described by absolute (*n*) and relative (%) frequencies. * Baseline data (T0).

**Table 2:** Dietary characteristics of participants at T2 and at T5 and mean change over a 3-year follow-up (studied sample *n* = 238).

	Median T2 (P25–P75)	Median T5 (P25–P75)	Diff ^1^	*p*-Value
**Total energy intake (kcal/day)**	1767.9 (1439.0–2071.0)	1615.4 (1264.3–2050.2)	−159.4	0.002 ^§^
**Macronutrients**				
**(% in relation to the calorie intake)**				
Proteins	18.3 (16.7–20.3)	18.6 (16.4–21.0)	0.3	0.124
Carbohydrates	34.8 (30.7–39.4)	32.7 (28.5–37.9)	−2.0	<0.001 ^§^
Lipids	41.6 (37.7–45.5)	42.4 (37.7–46.5)	0.8	0.052
Saturated fatty acids	15.6 (14.2–17.5)	16.4 (14.1–18.6)	0.5	0.038 ^§^
Polyunsaturated fatty acids	6.0 (5.1–7.2)	6.0 (4.9–8.0)	0.0	0.413
Omega 3 fatty acids	0.8 (0.7–1.0)	0.8 (0.6–1.0)	−0.0	0.065
Omega 6 fatty acids	4.7 (4.0–5.9)	4.8 (3.9–6.5)	0.0	0.238
Monounsaturated fatty acids	15.9 (13.9–18.3)	16.2 (13.8–18.5)	0.5	0.307
**Micronutrients (per 1000 kcal)**				
Vitamin D (µg/day)	1.3 (1.0–1.8)	1.3 (1.0–1.9)	0.0	0.226
Vitamin A (µg/day)	497.0 (409.6–565.3)	479.4 (391.9–569.1)	−13.4	0.530
Vitamin E (mg/day)	5.8 (4.9–7.0)	5.6 (4.7–7.2)	−0.0	0.770
Vitamin C (mg/day)	51.4 (37.2–71.7)	49.5 (35.8–65.7)	−0.6	0.768
Vitamin K (µg/day)	70.8 (54.9–85.6)	67.0 (52.5–88.5)	−2.7	0.176
Iron (mg/day)	8.3 (7.1–9.8)	7.8 (6.7–8.7)	−0.5	<0.001 ^§^
Calcium (mg/day)	508.8 (435.6–602.6)	481.0 (390.0–572.1)	−33.5	0.005 ^§^
Sodium (mg/day)	1512.8 (1360.1–1720.9)	1374.5 (1232.3–1514.8)	141.5	<0.001 ^§^
Potassium (mg/day)	1823.7 (1620.1–2030.7)	1820.0 (1608.8–2047.0)	−11.4	0.975
Magnesium (mg/day)	263.0 (220.4–308.3)	236.3 (195.0–275.7)	−22.0	<0.001 ^§^
Phosphorus (mg/day)	730.8 (653.1–782.7)	727.1 (643.0–794.4)	−2.3	0.834
Zinc (mg/day)	6.6 (5.9–7.4)	6.4 (5.6–7.2)	−0.2	0.004 ^§^

^1^ Median of the absolute difference between T2 and T5. ^§^ Results for which the *p*-value was statistically significant (<0.05).

**Table 3:** Baseline associations between macro- and micronutrient consumption and muscle health components.

Muscle Parameters at T2	Gait Speed		Muscle Strength	
Intake at T2	β	*p*-Value *	β	*p*-Value *
**Macronutrients**				
Calorie	5.538 × 10^−5^	0.122	0.003	0.003 ^§^
Protein	0.003	0.606	0.26	0.098
Carbohydrate	0.000	0.898	−0.077	0.280
Lipid	0.000	0.882	0.046	0.524
Saturated fatty acids	0.002	0.666	−0.031	0.828
Polyunsaturated fatty acids	−0.006	0.480	0.116	0.599
Omega 3 fatty acids	−0.056	0.230	2.578	0.031 ^§^
Omega 6 fatty acids	−0.006	0.562	0.015	0.951
Monounsaturated fatty acids	0.002	0.610	0.120	0.311
**Micronutrients**				
Vitamin D	−0.013	0.419	0.899	0.031 ^§^
Vitamin A	0.000	0.291	0.006	0.045 ^§^
Vitamin E	−0.009	0.373	0.119	0.630
Vitamin C	0.001	0.403	0.019	0.276
Vitamin K	0.001	0.247	0.042	0.013 ^§^
Iron	−0.005	0.552	−0.179	0.427
Calcium	−9.261 × 10^−5^	0.463	0.004	0.231
Sodium	6.525 × 10^−5^	0.221	0.002	0.176
Potassium	5.335 × 10^−5^	0.316	0.003	0.035 ^§^
Magnesium	−3.843 × 10^−5^	0.987	0.005	0.376
Phosphorus	−5.244 × 10^−5^	0.744	0.005	0.207
Zinc	0.006	0.585	0.119	0.685

* p-values obtained from linear regression including age, sex, BMI, number of drugs, number of concomitant diseases, physical activity level, smoking status, and kcal consumed at T2 as covariates. ^§^ Results for which the *p*-value was statistically significant (<0.05)

**Table 4:** Association between longitudinal changes in macro- and micronutrient consumption and longitudinal changes in muscle health components.

Change of Muscle Health Components between T2 and T5	Gait Speed		Muscle Strength	
Change of Consumption between T2 and T5	β	*p*-Value *	β	*p*-Value *
**Macronutrients**				
Calorie	1.645 × 10^−5^	0.506	4.612 × 10^−6^	0.994
Protein	0.004	0.326	−0.112	0.245
Carbohydrate	−2.795 × 10^−5^	0.988	−0.079	0.077
Lipid	0.001	0.513	0.077	0.077
Saturated fatty acids	0.003	0.375	0.183	0.051
Polyunsaturated fatty acids	0.001	0.875	0.013	0.927
Omega 3 fatty acids	−0.024	0.567	−0.206	0.838
Omega 6 fatty acids	0.001	0.814	0.024	0.873
Monounsaturated fatty acids	0.001	0.741	0.139	0.086
**Micronutrients**				
Vitamin D	−0.009	0.550	−0.485	0.164
Vitamin A	−7.567 × 10^−5^	0.300	0.000	0.904
Vitamin E	−0.003	0.621	0.024	0.877
Vitamin C	0.000	0.789	0.016	0.141
Vitamin K	0.000	0.631	0.015	0.174
Iron	−0.011	0.122	−0.260	0.112
Calcium	0.000	0.158	−0.001	0.792
Sodium	−5.914 × 10^−5^	0.230	−0.001	0.620
Potassium	−1.716 × 10^−5^	0.655	0.001	0.445
Magnesium	0.000	0.212	−0.002	0.628
Phosphorus	0.000	0.066	−0.005	0.111
Zinc	0.003	0.789	−0.274	0.232

* *p*-values obtained from linear regression including age, sex, BMI, smoking status, number of drugs, number of concomitant diseases, physical activity level, kcal consumed at T2, and muscle parameters value at T2 as covariates.

## Appendix A

**Table A1:** Dietary consumption at T2 and T5 (absolute values of consumption).

	Median T2 (P25–P75)	Median T5 (P25–P75)	Median of the Difference between T2 and T5	*p*-Value
**Total energy intake (kcal/day)**	1767.88 (1439.02–2070.96)	1615.41 (1264.34–2050.16)	−151.97	0.002
**Macronutrients**				
Proteins (g/day)	80.95 (64.42–97.07)	73.88 (54.54–101.21)	−4.15	0.020
Carbohydrates (g/day)	151.41 (124.52–180.36)	133.15 (100.49–162.91)	−18.64	<0.001
Lipids (g/day)	80.25 (61.86–98.12)	75.78 (57.35–98.67)	−5.30	0.083
Saturated fatty acids (g/day)	29.84 (22.99–38.51)	29.20 (21.39–37.94)	−1.51	0.121
Polyunsaturated fatty acids (g/day)	11.70 (8.65–15.47)	11.17 (7.96–15.61)	−0.64	0.188
Omega 3 (g/day)	1.56 (1.17–2.08)	1.40 (1.04–1.96)	−0.17	0.004
Omega 6 (g/day)	9.30 (6.85–12.25)	9.12 (6.37–12.90)	−0.38	0.299
Monounsaturated fatty acids (g/day)	30.84 (25.14–38.63)	29.26 (21.84–39.11)	−2.57	0.048
**Micronutrients**				
Sodium (mg/day)	2686.85 (2148.48–3228.33)	2190.19 (1717.06–2711.49)	−467.19	<0.001
Potassium (mg/day)	3210.88 (2615.01–3857.28)	2964.15 (2328.73–3685.18)	−249.92	<0.001
Magnesium (mg/day)	449.30 (362.82–575.20)	385.85 (307.86–467.27)	−69.45	<0.001
Phosphorus (mg/day)	1249.66 (1027.50–1543.91)	1181.27 (871.23–1524.57)	−79.40	0.002
Iron (mg/day)	14.53 (11.30–18.34)	12.43 (9.93–15.68)	−1.80	<0.001
Calcium (mg/day)	884.41 (703.09–1122.55)	784.78 (575.14–1045.49)	−91.62	<0.001
Zinc (mg/day)	11.60 (9.04–14.56)	10.28 (7.80–13.08)	−1.20	<0.001
Vitamin D (µg/day)	2.32 (1.63–3.17)	2.29 (1.54–3.49)	−0.04	0.657
Vitamin A (µg/day)	880.88 (644.14–1097.42)	789.27 (550.44–1047.74)	−66.28	0.006
Vitamin E (mg/day)	10.04 (7.78–13.29)	9.43 (7.18–12.68)	−0.31	0.037
Vitamin C (mg/day)	87.79 (62.72–126.79)	83.44 (57.71–112.70)	−6.14	0.010
Vitamin K (µg/day)	120.08 (92.41–163.89)	104.02 (74.77–151.08)	−9.92	0.003

**Table A2:** Association between longitudinal changes in macro- and micronutrient consumption and longitudinal relative changes in muscle health components.

Change of Muscle Health Components between T2 and T5	Gait Speed		Muscle Strength	
Change of Consumption between T2 and T5	β	*p*-Value	β	*p*-Value
Calorie	1.621 × 10^−5^	0.512	2.527 × 10^−6^	0.997
Protein	0.004	0.343	−0.113	0.235
Carbohydrate	−9.7219 × 10^−5^	0.961	−0.085	0.058
Lipid	0.001	0.481	0.083	0.059
Saturated fatty acids	0.004	0.356	0.195	0.039
Polyunsaturated fatty acids	0.001	0.846	0.024	0.864
Omega-3 fatty acids	−0.024	0.565	−2.220	0.826
Omega-6 fatty acids	0.002	0.784	0.037	0.806
Monounsaturated fatty acids	0.001	0.708	0.146	0.071
Vitamin D	−0.009	0.546	−0.486	0.162
Vitamin A	−7.492 × 10^−5^	0.307	0.000	0.846
Vitamin E	−0.003	0.646	0.036	0.816
Vitamin C	0.000	0.797	0.015	0.146
Vitamin K	0.000	0.623	0.014	0.183
Iron	−0.011	0.110	−0.282	0.087
Calcium	0.000	0.154	−0.001	0.801
Sodium	−6.001 × 10^−5^	0.224	−0.001	0.583
Potassium	−1.691 × 10^−5^	0.660	0.001	0.444
Magnesium	0.000	0.199	−0.002	0.548
Phosphorus	0.000	0.068	−0.005	0.106
Zinc	0.002	0.816	−0.290	0.204

** p*-values obtained from linear regression including age, sex, BMI, smoking status, number of drugs, number of concomitant diseases, physical activity (Minnesota), kcal consumed at T2, and muscle parameters value at T2.

**Table A3:** Adequation of the consumption of macro- and micronutrients at T2 and T5.

	Population with an Adequate Intake at T2 (%)	Population with an Adequate Intake at T5 (%)	*p*-Value
**Macronutrients**			
Proteins	90.3	79.4	0.001
Carbohydrates	1.3	0.8	0.625
Lipids	46.2	45.4	0.039
Saturated fatty acids	20.2	31.1	0.003
Polyunsaturated fatty acids	45.0	40.3	0.417
Omega-3 fatty acids	15.1	14.7	0.538
Omega-6 fatty acids	43.3	38.2	0.210
Monounsaturated fatty acids	69.7	55.9	0.006
**Micronutrients**			
Vitamin D	0.8	0.0	0.485
Vitamin A	85.7	79.8	0.059
Vitamin E	60.1	54.6	0.193
Vitamin C	46.2	42.4	0.391
Vitamin K	3.8	4.2	0.644
Iron	97.1	94.5	0.210
Calcium	69.3	56.7	0.001
Sodium	0.8	5.5	0.007
Potassium	29.8	30.7	0.002
Magnesium	95.8	85.7	<0.001
Phosphorus	97.5	94.5	0.092
Zinc	82.4	76.5	0.070

The prevalence of participants below the recommended daily allowance (RDA) for macronutrients, measured from the Belgian nutritional recommendations [53], and below the estimated average requirement (EAR) for micronutrients were computed. Since the EAR was not available in the Belgian nutritional recommendations, the following formulas were used [73,74]:

- For vitamins D, A, E, K, iron, calcium, sodium, potassium, phosphorus, and zinc: 0.77 * RDA
- For magnesium and vitamin C: 0.83 * RDA

It was established that the EAR is a reliable estimate of the usual intake requirements of a group of subjects [75].

RDA tailored to populations aged over 60 years where applied when available. Alternatively, recommendations for adults older than 18 years were used.

## References

[1] Ponti F., Santoro A., Mercatelli D., Gasperini C., Conte M., Martucci M., Sangiorgi L., Franceschi C., Bazzocchi A. (2020). Aging and Imaging Assessment of Body Composition: From Fat to Facts. Front. Endocrinol..

[2] Tangen G.G., Robinson H.S. (2020). Measuring physical performance in highly active older adults: Associations with age and gender?. Aging Clin. Exp. Res..

[3] Cruz-Jentoft A.J., Bahat G., Bauer J., Boirie Y., Bruyère O., Cederholm T., Cooper C., Landi F., Rolland Y., Sayer A.A. (2019). Sarcopenia: Revised European consensus on definition and diagnosis. Age Ageing.

[4] Auyeung T.W., Lee S.W.J., Leung J., Kwok T., Woo J. (2014). Age-associated decline of muscle mass, grip strength and gait speed: A 4-year longitudinal study of 3018 community-dwelling older Chinese. Geriatr. Gerontol. Int..

[5] Goodpaster B.H., Park S.W., Harris T.B., Kritchevsky S.B., Nevitt M., Schwartz A.V., Simonsick E.M., Tylavsky F.A., Visser M., Newman A.B. (2006). The Loss of Skeletal Muscle Strength, Mass, and Quality in Older Adults: The Health, Aging and Body Composition Study. J. Gerontol..

[6] Kuo H.-K., Leveille S.G., Yen C.-J., Chai H.-M., Chang C.-H., Yeh Y.-C., Yu Y.-H., Bean J.F. (2006). Exploring How Peak Leg Power and Usual Gait Speed Are Linkedto Late-Life Disability: Data from the National Health and Nutrition Examination Survey (NHANES), 1999-2002. Am. J. Phys. Med. Rehabil..

[7] Hardy S.E., Perera S., Roumani Y.F., Chandler J.M., Studenski S.A. (2007). Improvement in usual gait speed predicts better survival in older adults. J. Am. Geriatr. Soc..

[8] Roberts H.C., Syddall H.E., Cooper C., Aihie sayer A. (2012). Is grip strength associated with length of stay in hospitalised older patients admitted for rehabilitation? Findings from the Southampton grip strength study. Age Ageing.

[9] Leong D.P., Teo K.K., Rangarajan S., Lopez-Jaramillo P., Avezum A., Orlandini A., Seron P., Ahmed S.H., Rosengren A., Kelishadi R. (2015). Prognostic value of grip strength: Findings from the Prospective Urban Rural Epidemiology (PURE) study. Lancet.

[10] Schmid A., Duncan P.W., Studenski S., Lai S.M., Richards L., Perera S., Wu S.S. (2007). Improvements in speed-based gait classifications are meaningful. Stroke.

[11] De Rekeneire N., Visser M., Peila R., Nevitt M.C., Cauley J.A., Tylavsky F.A., Simonsick E.M., Harris T.B. (2003). Is a Fall Just a Fall: Correlates of Falling in Healthy Older Persons. The Health, Aging and Body Composition Study. J. Am. Geriatr. Soc..

[12] Studenski S., Perera S., Wallace D., Chandler J.M., Duncan P.W., Rooney E., Fox M., Guralnik J.M. (2003). Physical Performance Measures in the Clinical Setting. J. Am. Geriatr. Soc..

[13] Penninx B.W.J.H., Ferrucci L., Leveille S.G., Rantanen T., Pahor M., Guralnik J.M. (2000). Lower Extremity Performance in Nondisabled Older Persons as a Predictor of Subsequent Hospitalization. J. Gerontol..

[14] Hajek A., Brettschneider C., Eisele M., Kaduszkiewicz H., Mamone S., Wiese B., Weyerer S., Werle J., Fuchs A., Pentzek M. (2020). Correlates of hospitalization among the oldest old: Results of the AgeCoDe–AgeQualiDe prospective cohort study. Aging Clin. Exp. Res..

[15] Mijnarends D.M., Luiking Y.C., Halfens R.J.G., Evers S.M.A.A., Lenaerts E.L.A., Verlaan S., Wallace M., Schols J.M.G.A., Meijers J.M.M. (2018). Muscle, Health and Costs: A Glance at their Relationship. J. Nutr. Health Aging.

[16] Guerra R.S., Amaral T.F., Sousa A.S., Pichel F., Restivo M.T., Ferreira S., Fonseca I. (2015). Handgrip strength measurement as a predictor of hospitalization costs. Eur. J. Clin. Nutr..

[17] Tieland M., Trouwborst I., Clark B.C. (2018). Skeletal muscle performance and ageing. J. Cachexia Sarcopenia Muscle.

[18] Landi F., Camprubi-Robles M., Bear D.E., Cederholm T., Malafarina V., Welch A.A., Cruz-Jentoft A.J. (2019). Muscle loss: The new malnutrition challenge in clinical practice. Clin. Nutr..

[19] Ramsey K.A., Meskers C.G.M., Trappenburg M.C., Verlaan S., Reijnierse E.M., Whittaker A.C., Maier A.B. (2020). Malnutrition is associated with dynamic physical performance. Aging Clin. Exp. Res..

[20] Corcoran C., Murphy C., Culligan E.P., Walton J., Sleator R.D. (2019). Malnutrition in the elderly. Sci. Prog..

[21] Volkert D., Beck A.M., Cederholm T., Cereda E., Cruz-Jentoft A., Goisser S., de Groot L., Großhauser F., Kiesswetter E., Norman K. (2019). Management of Malnutrition in Older Patients—Current Approaches, Evidence and Open Questions. J. Clin. Med..

[22] Adly N.N., Abd-El-Gawad W.M., Abou-Hashem R.M. (2020). Relationship between malnutrition and different fall risk assessment tools in a geriatric in-patient unit. Aging Clin. Exp. Res..

[23] Roy M., Gaudreau P., Payette H. (2016). A scoping review of anorexia of aging correlates and their relevance to population health interventions. Appetite.

[24] Landi F., Lattanzio F., Dell’Aquila G., Eusebi P., Gasperini B., Liperoti R., Belluigi A., Bernabei R., Cherubini A. (2013). Prevalence and potentially reversible factors associated with anorexia among older nursing home residents: Results from the ulisse project. J. Am. Med. Dir. Assoc..

[25] Jadczak A.D., Visvanathan R. (2019). Anorexia of Aging—An Updated Short Review. J. Nutr. Health Aging.

[26] Landi F., Calvani R., Tosato M., Martone A.M., Ortolani E., Savera G., Sisto A., Marzetti E. (2016). Anorexia of aging: Risk factors, consequences, and potential treatments. Nutrients.

[27] Volkert D., Kiesswetter E., Cederholm T., Donini L.M., Eglseer D., Norman K., Schneider S.M., Ströbele-Benschop N., Torbahn G., Wirth R. (2019). Development of a Model on Determinants of Malnutrition in Aged Persons: A MaNuEL Project. Gerontol. Geriatr. Med..

[28] Ter Borg S., Verlaan S., Hemsworth J., Mijnarends D.M., Schols J.M.G.A., Luiking Y.C., De Groot L.C.P.G.M. (2015). Micronutrient intakes and potential inadequacies of community-dwelling older adults: A systematic review. Br. J. Nutr..

[29] Jensen G.L., Cederholm T. (2018). The malnutrition overlap syndromes of cachexia and sarcopenia: A malnutrition conundrum. Am. J. Clin. Nutr..

[30] Beaudart C., Sanchez-Rodriguez D., Locquet M., Reginster J.Y., Lengelé L., Bruyère O. (2019). Malnutrition as a strong predictor of the onset of sarcopenia. Nutrients.

[31] Amarya S., Singh K., Sabharwal M. (2015). Changes during aging and their association with malnutrition. J. Clin. Gerontol. Geriatr..

[32] Deutz N.E.P., Ashurst I., Ballesteros M.D., Bear D.E., Cruz-Jentoft A.J., Genton L., Landi F., Laviano A., Norman K., Prado C.M. (2019). The Underappreciated Role of Low Muscle Mass in the Management of Malnutrition. J. Am. Med. Dir. Assoc..

[33] Beaudart C., Locquet M., Touvier M., Reginster J.Y., Bruyère O. (2019). Association between dietary nutrient intake and sarcopenia in the SarcoPhAge study. Aging Clin. Exp. Res..

[34] McLean R.R., Mangano K.M., Hannan M.T., Kiel D.P., Sahni S. (2016). Dietary Protein Intake Is Protective Against Loss of Grip Strength Among Older Adults in the Framingham Offspring Cohort. J. Gerontol. Ser. A Biol. Sci. Med. Sci..

[35] Granic A., Mendonça N., Sayer A.A., Hill T.R., Davies K., Adamson A., Siervo M., Mathers J.C., Jagger C. (2018). Low protein intake, muscle strength and physical performance in the very old: The Newcastle 85+ Study. Clin. Nutr..

[36] Visser M., Deeg D.J.H., Lips P. (2003). Low Vitamin D and High Parathyroid Hormone Levels as Determinants of Loss of Muscle Strength and Muscle Mass (Sarcopenia): The Longitudinal Aging Study Amsterdam. J. Clin. Endocrinol. Metab..

[37] Houston D.K., Tooze J.A., Neiberg R.H., Hausman D.B., Johnson M.A., Cauley J.A., Bauer D.C., Cawthon P.M., Shea M.K., Schwartz G.G. (2012). 25-hydroxyvitamin D status and change in physical performance and strength in older adults. Am. J. Epidemiol..

[38] Fingeret M., Vollenweider P., Marques-Vidal P. (2019). No association between vitamin C and E supplementation and grip strength over 5 years: The Colaus study. Eur. J. Nutr..

[39] Granic A., Mendonça N., Hill T.R., Jagger C., Stevenson E.J., Mathers J.C., Sayer A.A. (2018). Nutrition in the very old. Nutrients.

[40] Giezenaar C., Chapman I., Luscombe-Marsh N., Feinle-Bisset C., Horowitz M., Soenen S. (2016). Ageing is associated with decreases in appetite and energy intake—A meta-analysis in healthy adults. Nutrients.

[41] JafariNasabian P., Inglis J.E., Reilly W., Kelly O.J., Ilich J.Z. (2017). Aging human body: Changes in bone, muscle and body fat with consequent changes in nutrient intake. J. Endocrinol..

[42] Peters D.M., Fritz S.L., Krotish D.E. (2013). Assessing the reliability and validity of a shorter walk test compared with the 10-Meter Walk Test for measurements of gait speed in healthy, older adults. J. Geriatr. Phys. Ther..

[43] Schaap L.A., Fox B., Henwood T., Bruyère O., Reginster J.Y., Beaudart C., Buckinx F., Roberts H., Cooper C., Cherubini A. (2016). Grip strength measurement: Towards a standardized approach in sarcopenia research and practice. Eur. Geriatr. Med..

[44] Arnault N. (2013). Table de Composition des Aliments, étude NutriNet-Santé. [Food Composition Table, NutriNet-Santé Study].

[45] Taylor H.L., Jacobs D.R., Schucker B., Knudsen J., Leon A.S., Debacker G. (1978). A questionnaire for the assessment of leisure time physical activities. J. Chronic Dis..

[46] Vetrano D.L., Landi F., Volpato S., Corsonello A., Meloni E., Bernabei R., Onder G. (2014). Association of sarcopenia with short- and long-term mortality in older adults admitted to acute care wards: Results from the CRIME study. J. Gerontol. Ser. A Biol. Sci. Med. Sci..

[47] Steffl M., Bohannon R.W., Petr M., Kohlikova E., Holmerova I. (2015). Relation between cigarette smoking and sarcopenia: Meta-analysis. Physiol. Res..

[48] Dallongeville J., Maré N., Fruchart J.-C., Amouyel P. (1998). Community and International Nutrition Cigarette Smoking Is Associated with Unhealthy Patterns of Nutrient Intake: A Meta-analysis. J. Nutr..

[49] Zadak Z., Hyspler R., Ticha A., Vlcek J. (2013). Polypharmacy and malnutrition. Curr. Opin. Clin. Nutr. Metab. Care.

[50] Streicher M., van Zwienen-Pot J., Bardon L., Nagel G., Teh R., Meisinger C., Colombo M., Torbahn G., Kiesswetter E., Flechtner-Mors M. (2018). Determinants of Incident Malnutrition in Community-Dwelling Older Adults: A MaNuEL Multicohort Meta-Analysis. J. Am. Geriatr. Soc..

[51] Beaudart C., Reginster J.Y., Petermans J., Gillain S., Quabron A., Locquet M., Slomian J., Buckinx F., Bruyère O. (2015). Quality of life and physical components linked to sarcopenia: The SarcoPhAge study. Exp. Gerontol..

[52] Thiébaut A., Kesse E., Com-Nougué C., Clavel-Chapelon F., Bénichou J. (2004). Ajustement sur l’apport énergétique dans l’évaluation des facteurs de risque alimentaires Adjustment for energy intake in the assessment of dietary risk factors. Rev. Epidemiol. Sante Publique.

[53] Conseil Supérieur de la Santé (CSS) (2016). Recommandations Nutritionnelles Pour la Belgique 2016.

[54] Wysokiński A., Sobów T., Kłoszewska I., Kostka T. (2015). Mechanisms of the anorexia of aging—A review. Age.

[55] Bohannon R.W. (2019). Minimal clinically important difference for grip strength: A systematic review. Soc. Phys. Ther. Sci..

[56] Beaudart C., Rolland Y., Cruz-Jentoft A.J., Bauer J.M., Sieber C., Cooper C., Al-Daghri N., Araujo de Carvalho I., Bautmans I., Bernabei R. (2019). Assessment of Muscle Function and Physical Performance in Daily Clinical Practice: A position paper endorsed by the European Society for Clinical and Economic Aspects of Osteoporosis, Osteoarthritis and Musculoskeletal Diseases (ESCEO). Calcif. Tissue Int..

[57] Robinson S., Granic A., Sayer A.A. (2019). Nutrition and muscle strength, as the key component of sarcopenia: An overview of current evidence. Nutrients.

[58] Úbeda N., Achón M., Varela-Moreiras G. (2012). Omega 3 fatty acids in the elderly. Br. J. Nutr..

[59] Lauretani F., Semba R.D., Bandinelli S., Dayhoff-Brannigan M., Giacomini V., Corsi A.M., Guralnik J.M., Ferrucci L. (2008). Low Plasma Carotenoids and Skeletal Muscle Strength Decline Over 6 Years. J. Gerontol..

[60] Rondanelli M., Faliva M., Monteferrario F., Peroni G., Repaci E., Allieri F., Perna S. (2015). Novel insights on nutrient management of sarcopenia in elderly. Biomed Res. Int..

[61] Dupont J., Dedeyne L., Dalle S., Koppo K., Gielen E. (2019). The role of omega-3 in the prevention and treatment of sarcopenia. Aging Clin. Exp. Res..

[62] Houston D.K., Cesari M., Ferrucci L., Cherubini A., Maggio D., Bartali B., Johnson M.A., Schwartz G.G., Kritchevsky S.B. (2007). Association Between Vitamin D Status and Physical Performance: The InCHIANTI Study. J. Gerontol..

[63] Remelli F., Vitali A., Zurlo A., Volpato S. (2019). Vitamin D deficiency and sarcopenia in older persons. Nutrients.

[64] Shea M.K., Loeser R.F., Hsu F.C., Booth S.L., Nevitt M., Simonsick E.M., Strotmeyer E.S., Vermeer C., Kritchevsky S.B. (2016). Vitamin K Status and Lower Extremity Function in Older Adults: The Health Aging and Body Composition Study. J. Gerontol. Ser. A Biol. Sci. Med. Sci..

[65] Azuma K., Inoue S. (2019). Multiple modes of vitamin K actions in aging-related musculoskeletal disorders. Int. J. Mol. Sci..

[66] (1989). Clausen Torben; Everts Maria Regulation of the Na,K-pump in skeletal muscle. Int. Soc. Nephrol..

[67] McLeod J.C., Stokes T., Phillips S.M. (2019). Resistance exercise training as a primary countermeasure to age-related chronic disease. Front. Physiol..

[68] Deutz N.E.P., Bauer J.M., Barazzoni R., Biolo G., Boirie Y., Bosy-Westphal A., Cederholm T., Cruz-Jentoft A., Krznariç Z., Nair K.S. (2014). Protein intake and exercise for optimal muscle function with aging: Recommendations from the ESPEN Expert Group. Clin. Nutr..

[69] Kamo T., Ishii H., Suzuki K., Nishida Y. (2019). The impact of malnutrition on efficacy of resistance training in community-dwelling older adults. Physiother. Res. Int..

[70] Nilsson M.I., Mikhail A., Lan L., Carlo A.D., Hamilton B., Barnard K., Hettinga B.P., Hatcher E., Tarnopolsky M.G., Nederveen J.P. (2020). A five-ingredient nutritional supplement and home-based resistance exercise improve lean mass and strength in free-living elderly. Nutrients.

[71] Gropper S.S., Tappen R.M., Vieira E.R. (2019). Differences In Nutritional And Physical Health Indicators Among Older African Americans, European Americans, And Hispanic Americans. J. Nutr. Gerontol. Geriatr..

[72] Du K., Goates S., Arensberg M.B., Pereira S., Gaillard T. (2018). Prevalence of Sarcopenia and Sarcopenic Obesity Vary with Race/Ethnicity and Advancing Age. Divers. Equal. Health Care.

[73] Kalonji E., Sirot V., Noel L., Guerin T., Margaritis I., Leblanc J.-C. (2015). Nutritional Risk Assessment of Eleven Minerals and Trace Elements: Prevalence of Inadequate and Excessive Intakes from the Second French Total Diet Study. Eur. J. Nutr. Food Saf..

[74] (2015). Agence nationale de sécurité sanitaire de l’alimentation, de l’environnement et du travail (ANSES) Avis de l’ANSES, Saisine n°2012-SA-0142; 14 rue Pierre et Marie Curie, 94701 Maisons-Alfort Cedex. https://www.anses.fr/fr/system/files/NUT2012sa0142.pdf.

[75] Carriquiry A.L. (1998). Assessing the prevalence of nutrient inadequacy. Public Health Nutr..

